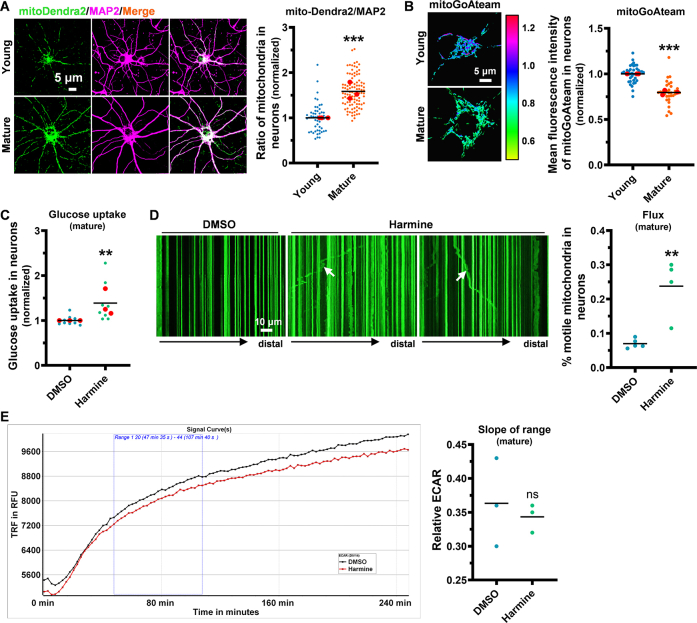# Harmine promotes axon regeneration through enhancing glucose metabolism

**DOI:** 10.1016/j.jbc.2025.108254

**Published:** 2025-02-02

**Authors:** Ruixuan Liu, Bing Zhou

**Affiliations:** 1 Beijing Advanced Innovation Center for Big Data-Based Precision Medicine, Beihang University, Beijing, China; 2 School of Biological Science and Medical Engineering, Beihang University, Beijing, China; 3 Interdisciplinary Innovation Institute of Medicine and Engineering Interdisciplinary, Beihang University, Beijing, China

**Keywords:** harmine, axon regeneration, glucose metabolism, metabolic coupling, mitochondrial function, energy supply, neuron

## Abstract

Axon regeneration requires a substantial mitochondrial energy supply. However, injured mature neurons often fail to regenerate due to their inability to meet these elevated energy demands. Our findings indicate that harmine compensates for the energy deficit following axonal injury by enhancing the coupling between glucose metabolism and mitochondrial homeostasis, thereby promoting axon regeneration. Notably, harmine facilitates mitochondrial biogenesis and enhances mitophagy, ensuring efficient mitochondrial turnover, and energy supply. Thus, harmine plays a crucial role in enhancing glucose metabolism to maintain mitochondrial function, demonstrating significant potential in treating mature neuronal axon injuries and sciatic nerve injuries.

Axon regeneration is crucial for restoring nervous system function, particularly in the treatment of neural injuries and degenerative diseases. Effective neuron regeneration can significantly enhance patients’ quality of life and mitigate long-term disability. Energy metabolism is essential for axon regeneration in injured neurons (1). Mitochondria, the energy factories, provide the necessary ATP for regenerative process, especially when metabolic demands increase significantly during axon regeneration (2, 3, 4). Importantly, axonal injury often results in mitochondrial dysfunction (5). Therefore, restoring mitochondrial function and maintaining energy supply are essential for promoting nerve regeneration. Previous studies have shown that nerve regeneration was associated with mitochondrial motility (6, 7, 8). However, the roles of energy metabolism, particularly glucose-mitochondria metabolism connection, and mitochondrial quality control in this process remain unclear.

Metabolic coupling, involving the interconnection between glycolysis, the tricarboxylic acid cycle, and mitochondrial oxidative phosphorylation, can meet cellular energy needs under various conditions. Some cancer cells increase the coupling of glycolysis and mitochondrial respiration, diverting glucose metabolic flux toward oxidative phosphorylation to promote cell proliferation (9). In the nervous system, enhancing the coupling of glucose metabolism and mitochondrial function in neurons facilitates bioenergetic metabolism and synapse formation, which are critical for improving cognitive function and neural plasticity (10). However, the exact mechanism of metabolic coupling during axonal regeneration is not clear.

Inhibitor of DNA binding 2 (ID2) is a protein induced under hypoxic conditions and has been identified as a key transcription factor that promotes axonal regeneration (11, 12). However, transduction strategies, such as delivery *via* adeno-associated virus, present limitations of immune response and delayed transgene expression in clinical applications. In comparison, small molecule drugs offer distinct advantages in the central nervous system due to their simpler control and application. Studies have demonstrated that harmine, a small compound, significantly upregulates the expression of ID2 protein in various cell types (13, 14), potentially playing a pivotal role in axon regeneration. Previous studies have demonstrated that harmine significantly contributes to promoting cell proliferation, osteogenesis, and maintaining neuron survival (15, 16, 17, 18, 19, 20). During early development, harmine reduces neurite numbers by inhibiting dual-specificity tyrosine-phosphorylation-regulated kinase 1A (DYRK1), while also promoting an increase in neurite length (21). Additionally, harmine has been found to inhibit mitochondrial complexes I, II, and IV (22), potentially inducing mitophagy. However, its effects on mitochondrial homeostasis during axon regeneration remain unclear.

Meeting the high energy demands required for axon regeneration following injury remained a significant challenge (4). Our research identified a novel modulator that enhanced the coupling of glucose metabolism with mitochondrial function. Specifically, we explored how harmine contributed to mitochondrial quality control, thereby sustaining energy supply and promoting axon regeneration. We concluded that strengthening glycolytic-mitochondria coupling was critical for axon regeneration. By conducting an in-depth study of the metabolic mechanisms of harmine in this process, our research provided a new paradigm for therapeutic interventions in neural repair.

## Results

### Harmine promotes axon regeneration in cortical neurons and DRG neurons

Polarization is a key process in neuronal development, where a single axon and multiple dendrites are formed based on neuronal differentiation, ultimately leading to the formation of highly specialized mature neurons (23). Notably, the extent of neuronal differentiation directly influences the capacity for axon regeneration (24). Specifically, neurons in the early stages of development, characterized by a lower degree of differentiation, exhibit robust axon regenerative potential. However, as neurons mature and differentiation advances, this regenerative capability diminishes. Based on this, inducing neuronal dedifferentiation which involves reverting neurons to an immature state may enhance their regenerative ability after injury.

**Figure 1:**
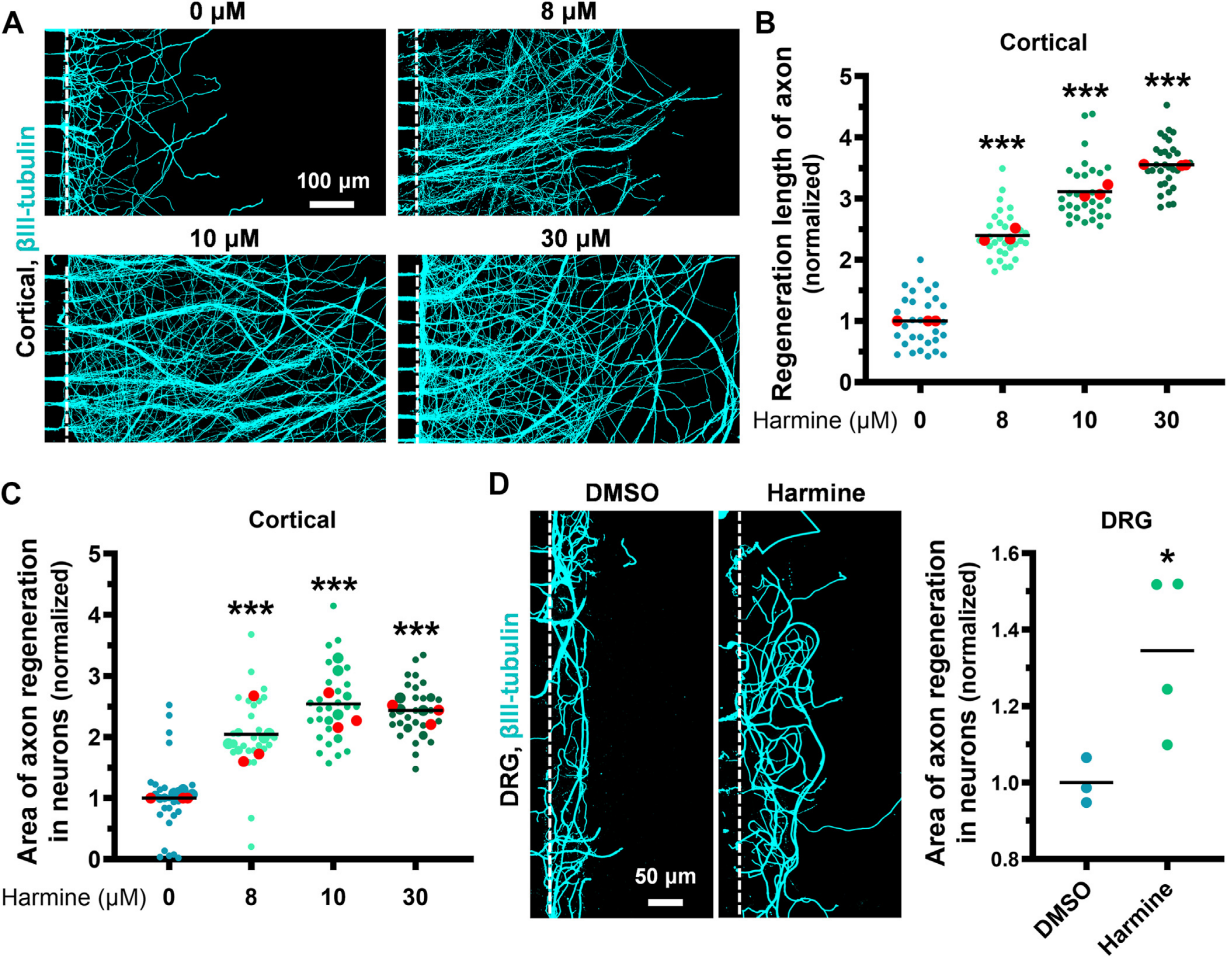
**Harmine promotes axon regeneration in neurons**. *A* and *B*, cortical neurons cultured in microfluidic devices underwent axotomy at DIV7, followed by treatment with varying concentrations of harmine (0, 8, 10, and 30 μM). After 48 h, neurons were stained for βIII-tubulin (*cyan*). Representative images (*A*) and quantitative analysis (*B*) of axonal regeneration lengths were normalized to the control group. (0 μM *versus* 8 μM: *p* < 0.001; 0 μM *versus* 10 μM: *p* < 0.001; 0 μM *versus* 30 μM: *p* < 0.001). N = 3; n_(0 μM)_ = 30, n_(8 μM)_ = 30, n_(10 μM)_ = 30, n_(30 μM)_ = 30. The scale bar represents 100 μm. *C*, quantitative analysis of axonal regeneration area in (*A*), normalized to the control group. (0 μM *versus* 8 μM: *p* < 0.001; 0 μM *versus* 10 μM: *p* < 0.001; 0 μM *versus* 30 μM: *p* < 0.001). N = 3; n_(0 μM)_ = 30, n_(8 μM)_ = 30, n_(10 μM)_ = 30, n_(30 μM)_ = 30. *D*, DRG neurons cultured in microfluidic devices underwent axotomy at DIV7, followed by treatment with 10 μM harmine for 48 h and βIII-tubulin staining (*left*, *cyan*). Axonal regeneration area was normalized to the control group (*right*). (DMSO *versus* Harmine: *p* = 0.0418). N_(DMSO)_ = 3, N_(Harmine)_ = 4. The scale bar represents 50 μm. Data are expressed as mean. Significance: ∗*p* < 0.05, ∗∗∗*p* < 0.001. (*B*) Mann-Whitney U-test. *C* and *D*, two-tailed unpaired Student’s *t* test. DRG, dorsal root ganglia; DMSO, dimethylsulfoxide; DIV, day *in vitro*.

**Figure S1:**
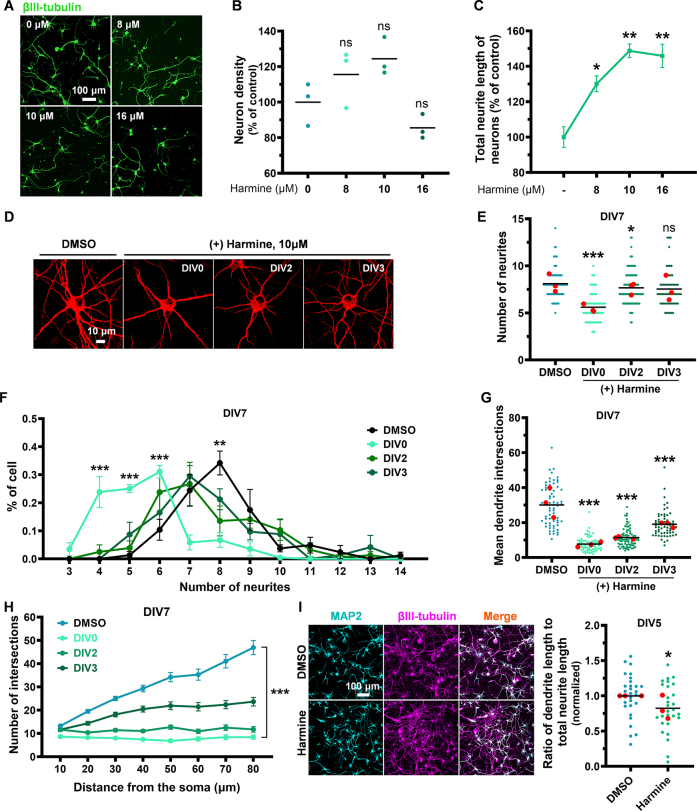


It has been reported that ID2 overexpression induced neuroblastoma dedifferentiation and enhanced chondrogenic proliferation (25, 26). Since harmine stabilizes ID2, we speculated that harmine could promote neuronal dedifferentiation. To test this, we first treated neurons with varying concentrations of harmine and assessed cell viability after 48 h, finding 10 μM to be optimal (Fig. 1, *A*–*C*). The number of neurites present in each cell serves as an indicator of differentiation in terms of neuronal polarity (27). To further verify the effect of harmine on neuronal polarity. We applied harmine at critical development stages for neuron polarity (day *in vitro*, DIV0, DIV2, and DIV3) and evaluated neuron branching (28). At the early stage (DIV0), harmine treatment significantly reduced the number of neuron branches, with most branches clustering between 4 and 6, while simultaneously enhancing axonal outgrowth (Fig. S1, *D*–*F*). In contrast, at the later stage (DIV3), harmine did not significantly affect the number of neuron branches (Fig. S1*E*). In addition, we performed Sholl analysis to evaluate the effect of harmine on neurite complexity in the neurodevelopmental paradigm. Our results demonstrated that harmine significantly reduced dendritic complexity during early neuronal development, suggesting that harmine may redirect intracellular resources toward axon elongation, thereby favoring axon regeneration over dendritic maintenance (Fig. S1, *G* and *H*). MAP2/βⅢ-tubulin to distinguish neuronal dendrites/axons in different assays, as MAP2 was specifically expressed in dendrites and cell bodies, whereas βⅢ-tubulin was used to visualize axons. Dendritic and axonal elaboration serves as a key indicator of neuronal maturation (29). Thus, we used MAP2 and βⅢ-tubulin antibodies to determine whether harmine affects neuronal maturity. Based on the MAP2/βⅢ-tubulin ratio, we found that harmine induces neuronal dedifferentiation in the later stages of neuronal polarity formation and reallocates resources to axons, suggesting it may promote axonal growth (Fig. S1*I*).

In prior research, harmine has shown neuroprotective effects following traumatic brain injury and regeneration of periodontal tissue (19, 20). Here, we sought to investigate the potential of harmine in promoting axon regeneration. We first conducted *in vitro* cultures of cortical and dorsal root ganglia (DRG) neurons. Our previous studies indicated that neurons cultured *in vitro* for 7 days (DIV7) were considered young and exhibited robust regenerative ability (6). In contrast, neurons cultured for 12 days or longer were regarded as mature and showed a significant decline in their ability to regenerate. Therefore, we have focused on using DIV7 neurons as the primary *in vitro* model to investigate the metabolic mechanisms underlying neuron regeneration. Using microfluidic devices, we established injury model that enables the compartmentalized culture of neuronal cell bodies and axons. Specifically, the axons of neurons cultured in the somatic chamber grew along the microchannels of the microfluidic device, reaching the opposite axon chamber at DIV7. To evaluate the capacity of harmine to promote axonal regeneration, we performed axotomy at DIV7 and subsequently cultured the cells for an additional 48 h in neurobasal medium with varying harmine concentrations. We found that harmine promoted axonal regeneration in cortical neurons in a dose-dependent manner, as shown by the quantitative assessment of regeneration length and area (Fig. 1, *A*–*C*). We also observed that harmine enhanced axon regeneration in DRG neurons 48 h following axonal injury (Fig. 1*D*).

Taken together, harmine reduces the dendritic complexity of neurons during early neural development, which allows it to redirect metabolites toward injured axons in the later phases for axonal regeneration.

### Harmine enhances axon regeneration *via* glycolytic-mitochondrial coupling

**Figure 2:**
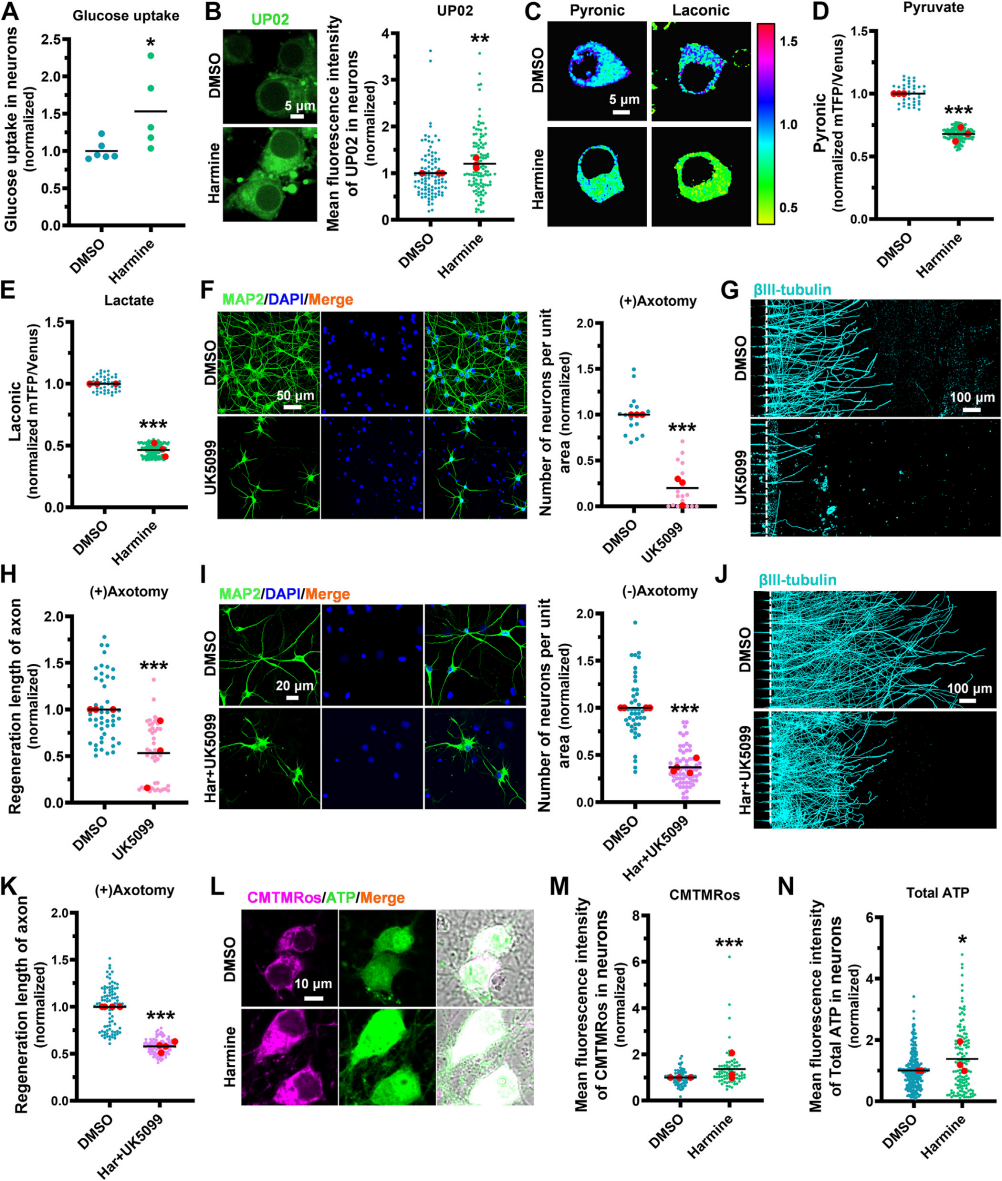
**Harmine enhances neuronal survival and axon regeneration *via* glycolytic–mitochondria coupling.***A*, measurement of glucose uptake in uninjured neurons (DIV7) treated with harmine using a glucose assay kit, normalized to the control group. (DMSO versus Harmine: *p* = 0.037). N_(DMSO)_ = 6, N_(Harmine)_ = 5. *B*, representative images (*left*) and quantitative analysis (*right*) of glucose uptake in uninjured neurons (DIV7) after 4 h of harmine treatment, stained with UP02 (dilution 1:500, 15 min), normalized to the control group. (DMSO versus Harmine: *p* = 0.0071). N = 3; n_(DMSO)_ = 97, n_(Harmine)_ = 116. UP02 (*green*). The scale bar represents 5 μm. *C*, fluorescence images of pyruvate (*left*) and lactate (*right*) levels in uninjured neurons (DIV7) labeled with lentiviral Pyronic and Laconic after treatment with harmine. Images were pseudocolored *via* the ratio of fluorescence emitted at 480 nm and 535 nm. The scale bar represents 5 μm. *D*, pyruvate levels in uninjured neurons (DIV7) labeled with lentiviral Pyronic and treated with harmine, showing a significant decrease in the treated group compared to control. (DMSO *versus* harmine: *p* < 0.001). N = 3; n_(DMSO)_ = 38, n_(Harmine)_ = 165. *E*, lactate levels in uninjured neurons (DIV7) infected with lentiviral Laconic and treated with harmine, showing a significant decrease in the treated group compared to control. (DMSO versus Harmine: *p* < 0.001). N = 3; n_(DMSO)_ = 44, n_(Harmine)_ = 197. *F*, representative images (*left*) and quantitative analysis (*right*) are shown for injured cortical neurons (DIV7) on the soma side of the microfluidic device after 24 h treatment with 10 μM UK5099. These data display the number of injured neurons per unit area, normalized to the control group. (DMSO *versus* UK5099: *p* < 0.001). N = 3; n_(DMSO)_ = 15, n_(UK5099)_ = 17. MAP2 (*green*), DAPI (*blue*). The scale bar represents 50 μm. *G* and *H*, quantification of axon regeneration length in cortical neurons cultured in microfluidic devices and subjected to axotomy at DIV7, treated with 10 μM UK5099 for 24 h, and stained with βIII-tubulin (*cyan*, *G*), normalized to the control group. The results showed that UK5099 inhibits axon regeneration (*H*). (DMSO *versus* UK5099: *p* < 0.001). N = 3; n_(DMSO)_ = 46, n_(UK5099)_ = 44. The scale bar represents 100 μm. *I*, representative images (*left*) of undamaged cortical neurons (DIV7) treated with 10 μM harmine and 10 μM UK5099, alongside quantitative analysis (*right*) of neuron density per unit area. (DMSO versus Har + UK5099: *p* < 0.001). N = 4; n_(DMSO)_ = 43, n_(UK5099)_ = 63. MAP2 (*green*), DAPI (*blue*). The scale bar represents 20 μm. *J* and *K*, representative images (*J*) and quantitative analysis (*K*) of axonal regeneration in injured neurons (DIV7) after 48 h of treatment with Harmine and UK5099 (Har + UK5099). (DMSO *versus* Har + UK5099: *p* < 0.001). N = 4; n_(DMSO)_ = 89, n_(UK5099)_ = 108. βⅢ-tubulin (*cyan*). The scale bar represents 100 μm. *L*-*N*, the mean fluorescence intensities of CMTMRos (*magenta*) and total ATP (*green*) in uninjured neurons were observed at DIV7 after 4 h of harmine treatment (*L*). Quantitative analysis revealed an increase in ΔΨ_m_ in neurons treated with harmine (*M*). (DMSO versus Harmine: *p* < 0.001). N = 3; n_(DMSO)_ = 80, n_(Harmine)_ = 71. Quantitative analysis showed that ATP level increased in neurons treated with harmine (*N*). (DMSO versus Harmine: *p* = 0.0363). N = 3; n_(DMSO)_ = 250, n_(Harmine)_ = 132. The scale bar represents 10 μm. Data are expressed as mean. Significance: ∗*p* < 0.05, ∗∗*p* < 0.01, ∗∗∗*p* < 0.001. *D*, *F*, *H*, and *I*, two-tailed unpaired Student’s *t* test. *A*, *B*, *E*, *K*, *M*, and *N*, Mann-Whitney U-test. DMSO, dimethylsulfoxide; DAPI, 4′,6-diamidino-2-phenylindole; DIV, day *in vitro*; ΔΨm, mitochondrial membrane potential.

**Figure S2:**
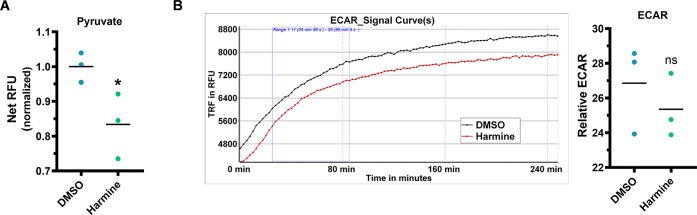


Glucose is the primary fuel for the neurons, and its coupling with mitochondrial metabolism allows cells to efficiently use available nutrients and energy sources for growth (30, 31). To determine the relationship between glucose and metabolic transformation, we first investigated the effect of harmine on neuronal glucose uptake. We found that glucose uptake significantly increased after harmine treatment, using a glucose uptake assay kit (Fig. 2*A*). To eliminate the interference of glial cells under culture conditions, we performed *in situ* UP02 fluorescence staining, a more specific glucose uptake dye that does not interfere with endogenous glucose metabolism on neurons. We further confirmed that harmine increased glucose uptake (Fig. 2*B*). Notably, harmine significantly reduced the pyruvate level, suggesting that harmine may facilitate the entry of pyruvate into mitochondria for further metabolism (Figs. 2, *C* and *D*; S2*A*). Furthermore, we observed that the lactate level decreased in neurons, while the extracellular acidification rate (ECAR) showed no significant changes (Figs. 2, *C* and *E*; S2*B*). These suggest that lactate is not released extracellularly but rather used within the cell after glucose uptake.

Rapidly proliferating cells, such as cancer cells, often exhibit the Warburg effect, which shifts glycolysis toward lactate production. In addition, tumor cells also maintain functional mitochondria that support various biosynthetic processes. Although still a subject of debate, the coupling between glycolysis and mitochondrial metabolism appears to enable tumor cells to maximize the utilization of nutrients and energy sources, which fuel their growth and expansion. However, it is not clear whether this coupling determines axonal regeneration. To provide evidence that glucose-mitochondrial coupling promotes axonal regeneration, we sought to use UK5099 to block the entry of glycolytic-derived pyruvate into mitochondria. The results demonstrate that this uncoupling treatment significantly reduces neuronal survival and inhibits axon regeneration (Fig. 2, *F*–*H*). Thus, metabolic coupling between glycolysis and mitochondria is crucial for axon regeneration. To further determine the relationship between harmine-induced glucose uptake increase, and neuronal survival, as well as axonal regeneration, we treated injured neurons with harmine in the presence of UK5099. In uninjured conditions, the cotreatment of harmine and UK5099 reduced neuronal survival (Fig. 2*I*). Similarly, in injured neurons, cotreatment with harmine and UK5099 significantly reduced regeneration length (Fig. 2, *J* and *K*). To specifically observe whether harmine is related to mitochondrial function recovery, we examined the mitochondrial membrane potential (ΔΨ_m_) and total ATP levels. We found that the mitochondrial membrane potential and total ATP levels in neurons significantly increased after harmine treatment (Fig. 2, *L*–*N*). Collectively, these results suggest that harmine promotes axon regeneration through enhancing glycolytic-mitochondria coupling.

**Figure 3:**
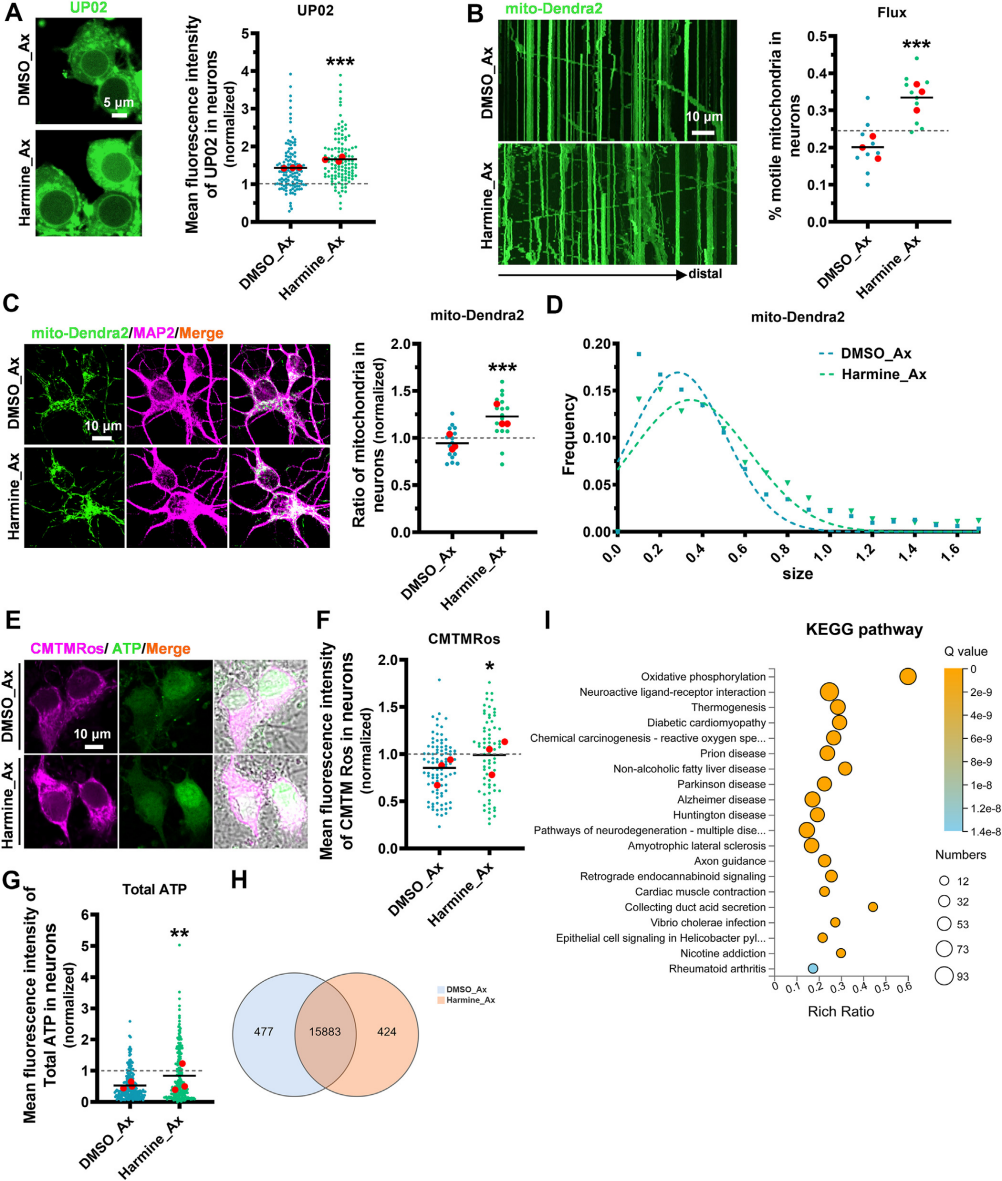
**Harmine promotes axon regeneration through glucose metabolism and mitochondrial function**. *A*, neurons at DIV7 were subjected to axotomy and treated with harmine for 4 h, followed by staining with UP02 (dilution 1:500, 15 min) for fluorescence images (*left*). Quantitative analysis of glucose uptake (*right*) was normalized to the uninjured control group. (DMSO_Ax *versus* Harmine_Ax: *p* < 0.001). N = 3; n_(DMSO_Ax)_ = 123, n_(Harmine_Ax)_ = 116. The scale bar represents 5 μm. *B*, kymographs (*left*) and quantitative analysis (*right*) show increased axonal mitochondrial transport in injured neurons after harmine treatment. Cortical neurons were infected with lentivirus mito-Dendra2. Time-lapse images were collected at DIV7 recorded for 100 frames at 5 s intervals. In the kymographs, *vertical lines* represent stationary mitochondria, and *arrows* indicate the direction of distal mitochondrial movement. (DMSO_Ax *versus* Harmine_Ax: *p* < 0.001). N = 3; n_(DMSO_Ax)_ = 9, n_(Harmine_Ax)_ = 10. The scale bar represents 10 μm. *C*, representative images (*left*) and quantitative analysis (*right*) of the ratio of mito-Dendra2-labeled mitochondria to neuron area in control (DMSO) and harmine-treated group (harmine) after axotomy at DIV7, normalized to the uninjured control group. (DMSO_Ax versus Harmine_Ax: *p* < 0.001). N = 3; n_(DMSO_Ax)_ = 15, n_(Harmine_Ax)_ = 16. mito-Dendra2 (*green*), MAP2 (*magenta*). The scale bar represents 10 μm. *D*, quantitative analysis of the Gaussian fitting curves of mitochondrial size distribution labeled with mito-Dendra2 in the control group (DMSO) and the harmine-treated group (Harmine) after axotomy at DIV7. N_(DMSO_Ax)_ = 18, N_(Harmine_Ax)_ = 18. *E*-*G*, representative images of CMTMRos (*magenta*) and ATP (*green*) in neurons from the DMSO_Ax and Harmine_Ax groups, following 4 h treatment with DMSO or harmine at DIV7 (*E*). Quantitative analysis showed increased ΔΨ_m_ production in harmine-treated injured neurons at DIV7 (*F*). (DMSO_Ax versus Harmine_Ax: *p* = 0.0118). N = 3; n_(DMSO_Ax)_ = 92, n_(Harmine_Ax)_ = 71. ATP levels were quantitatively analyzed in injured neurons from the DMSO_Ax and Harmine_Ax groups at DIV7 (G). (DMSO_Ax versus Harmine_Ax: *p* = 0.0019). N = 3; n_(DMSO_Ax)_ = 209, n_(Harmine_Ax)_ = 215. CMTMRos (*magenta*), ATP (*green*). The scale bar represents 10 μm. *H*, Venn diagram of gene expression levels in injured neurons (DIV7) treated with or without harmine. *I*, KEGG enrichment analysis of upregulated genes in injured neurons after harmine treatment. Data are expressed as mean. Significance: ∗*p* < 0.05, ∗∗*p* < 0.01, ∗∗∗*p* < 0.001. (*A*), (*G*) Mann-Whitney U-test. *B*, *C*, and *F* two-tailed unpaired Student’s *t* test. DMSO, dimethylsulfoxide; KEGG, Kyoto Encyclopedia of Genes and Genomes; DIV, day *in vitro*; ΔΨm, mitochondrial membrane potential.

**Figure S3:**
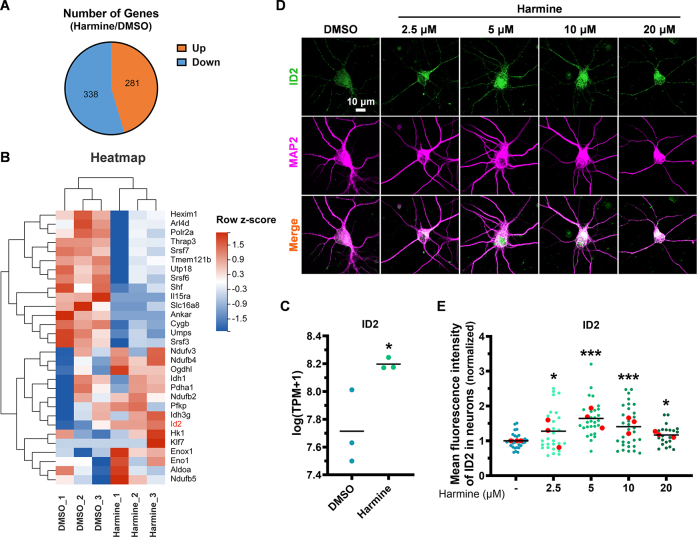


To evaluate the response genes of neurons treated with harmine, RNA-seq analysis was performed. Following 4 h of harmine treatment, 281 genes were upregulated and 338 genes were downregulated as compared with the control (Fig. S3*A*). A heatmap revealed decreased slc16a8 mRNA expression, indicating reduced lactate efflux, and downregulation of cytoglobin (Cygb), suggesting increased oxygen utilization (Fig. S3*B*) (32). Additionally, genes related to glycolysis and mitochondrial function, such as phosphofructokinase (Pfkp), hexokinase 1 (Hk1), isocitrate dehydrogenase 1 (Idh1), NADH:ubiquinone oxidoreductase subunit b2 (Ndufb2), and NADH:ubiquinone oxidoreductase subunit b4 (Ndufb4), were upregulated, indicating enhanced neuronal energy metabolism (Fig. S3*B*). As expected, ID2 mRNA showed a marked increase (Fig. S3*C*). To confirm harmine’s role as an ID2 stabilizer, we examined the effect of varying harmine concentrations on ID2 expression in neurons using immunofluorescence staining, which demonstrated a significant rise in ID2 protein levels (Fig. 3, *D* and *E*). Overall, these findings suggest that harmine enhances metabolic coupling by stabilizing ID2 protein and enables metabolic adaptation.

### Harmine promotes axon regeneration by enhancing glucose metabolism and maintaining mitochondrial function

**Figure S4:**
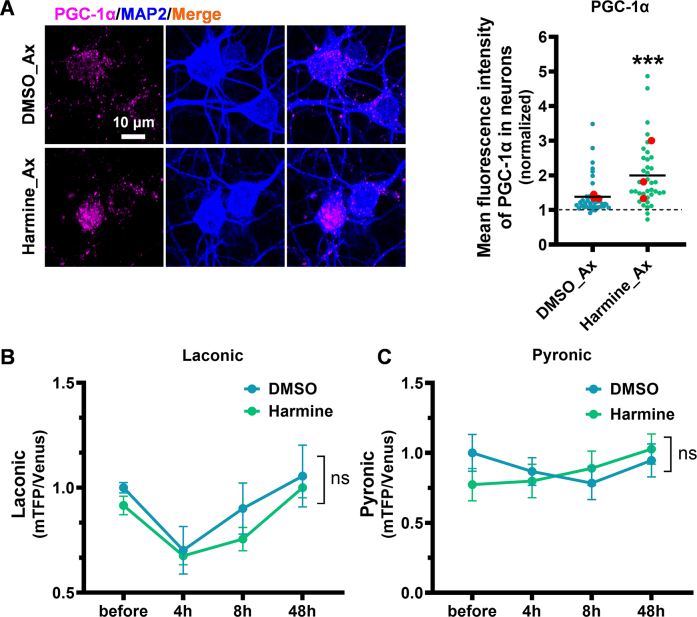


Nerve injuries typically result in mitochondrial dysfunction (5). To determine the metabolic mechanism by which harmine promotes axon regeneration, we analyzed glucose uptake 4 h after axon injury. Compared to the control group (dimethylsulfoxide, DMSO), harmine significantly enhanced glucose uptake in injured neurons (Fig. 3*A*). Our previous research demonstrated that mitochondria motility plays a major role in nerve regeneration (6). To this end, we labeled mitochondria with lentivirus mito-Dendra2 to further evaluate mitochondrial transport and distribution. We observed that harmine facilitated mitochondrial transport within axons, and increased both the proportion and size of mitochondria in neurons (Fig. 3, *B*–*D*). Peroxisome proliferator-activated receptor gamma coactivator 1-α (PGC-1α) is a key regulator of mitochondrial biogenesis and function (33, 34). Since our snapshot observation of the increased mitochondrial numbers by harmine may not explicitly explain how neuron regeneration is tied to metabolism, we further assessed PGC-1α levels to better explain the long-term regeneration promotion effects. Surprisingly, harmine increased PGC-1α protein expression in injured neurons 2 h post treatment (Fig. S4*A*). Moreover, both mitochondrial ΔΨ_m_ and total ATP levels in injured neurons significantly increased 4 h after harmine treatment, further confirming that harmine enhanced mitochondrial function (Fig. 3, *E*–*G*).

**Figure 4:**
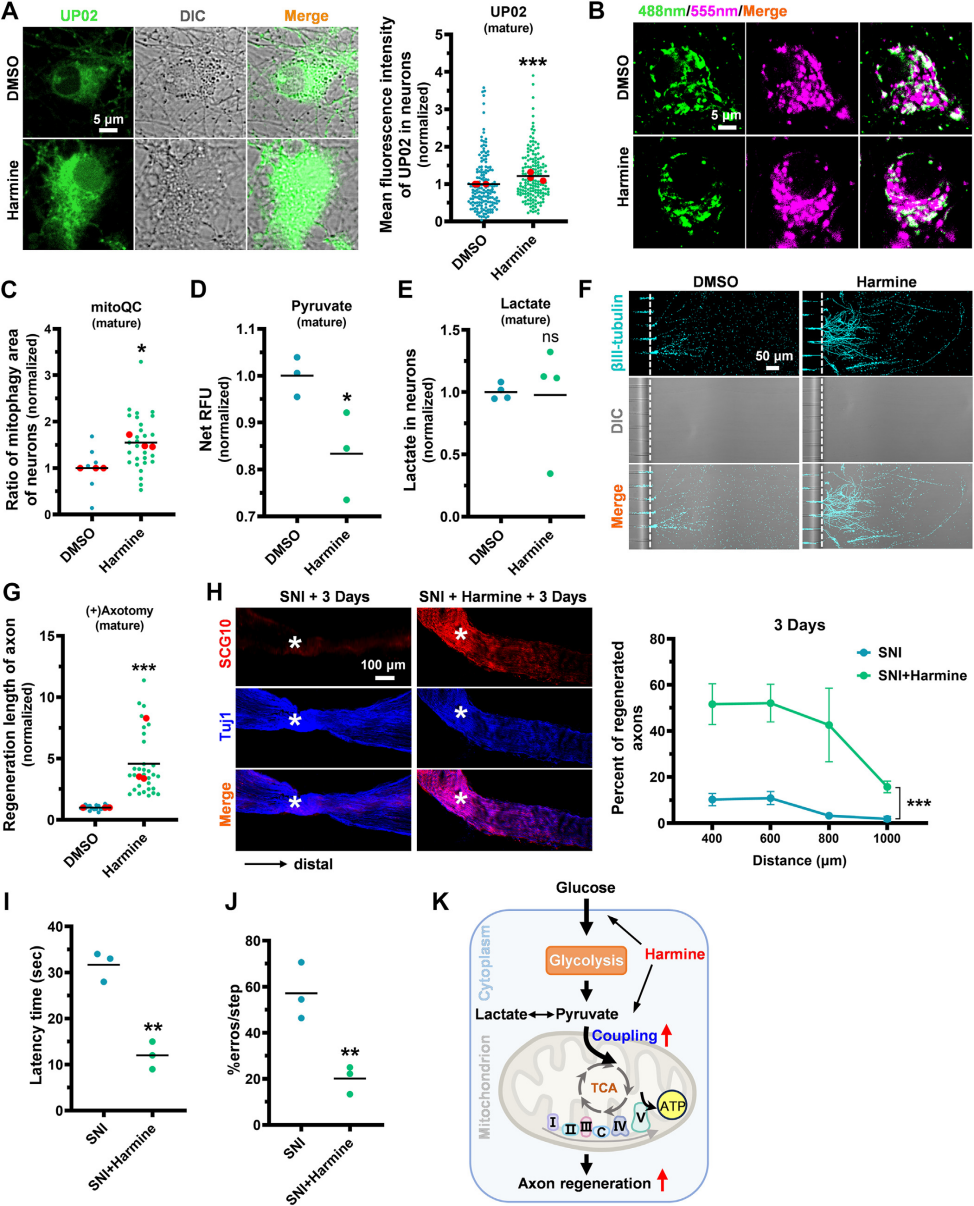
**Harmine enhances axonal regenerative ability and restores function to mature neurons**. *A*, representative images (*left*) and quantitative analysis (*right*) of glucose uptake in uninjured mature neurons after 4 h of harmine treatment at DIV30, normalized to the control group. (DMSO *versus* Harmine: *p* = 0.0002). N = 3; n_(DMSO)_ = 161, n_(Harmine)_ = 163. UP02 (*green*), DIC (*gray*). The scale bar represents 5 μm. *B*, neurons infected with lentivirus mitoQC show enhanced mitophagy in uninjured mature neurons (DIV30) 24 h after harmine treatment. Healthy mitochondria emit green fluorescence when excited at 488 nm and magenta fluorescence when excited at 555 nm, whereas mitochondria undergoing autophagy emit magenta fluorescence only when excited at 555 nm, due to the loss of green fluorescence caused by the acidic environment of lysosomes. The scale bar represents 5 μm. *C*, quantitative analysis from three independent experiments as described in (*B*). (DMSO versus Harmine: *p* = 0.0454). N = 3; n_(DMSO)_ = 6, n_(Harmine)_ = 28. *D*, pyruvate levels in uninjured neurons (DIV30) were measured using a pyruvate assay kit 4 h after harmine treatment, showing a reduction. (DMSO *versus* Harmine: *p* = 0.0487). N_(DMSO)_ = 3, N_(Harmine)_ = 3. *E*, lactate levels in uninjured neurons (DIV30) were measured using a lactate assay kit 4 h after DMSO or harmine treatment. (DMSO *versus* Harmine: *p* = 0.9198). N_(DMSO)_ = 4, N_(Harmine)_ = 4. *F*, mature neurons (DIV30) cultured in microfluidic devices underwent axotomy and were stained with βIII-tubulin 72 h later. βIII-tubulin (*cyan*), DIC (*gray*). The scale bar represents 50 μm. *G*, quantitative analysis of axonal regeneration length in injured mature neurons (DIV30) after 72 h of treatment with DMSO or Harmine in (*F*). *H*, representative images of sciatic nerves from control (SNI) and harmine-treated (SNI + Harmine) groups, stained with SCG10 to visualize regenerating axons 3 days after crush injury (left). The *asterisk* indicates the crush site. Percentage of regenerating axons at the specified distance distal to the lesion site (*right*). (SNI *versus* SNI + Harmine: *p* < 0.001). N_(SNI)_ = 3, N_(SNI + Harmine)_ = 3. SCG10 (*red*), Tuj1 (*blue*). The scale bar represents 100 μm. *I*, latency for 8-week-old mice with sciatic nerve injury, treated with control and harmine, to traverse the balance beam. The harmine group exhibited a significantly reduced traversal time, indicating improved motor coordination and balance. (SNI versus SNI + Harmine: *p* = 0.0015). N_(SNI)_ = 3, N_(SNI + Harmine)_ = 3. *J*, the walking error rate of mice (aged 8 weeks) with sciatic nerve injury treated with control and harmine. The harmine group showed a significantly lower error rate, reflecting enhanced motor function and balance control. (SNI *versus* SNI + Harmine: *p* = 0.0095). N_(SNI)_ = 3, N_(SNI + Harmine)_ = 3. *K*, diagram of harmine promoting regeneration through enhanced glucose uptake and coupled glycolysis with mitochondrial function. Data are expressed as mean. Significance: ∗*p* < 0.05, ∗∗*p* < 0.01, ∗∗∗*p* < 0.001; ns, not significant. (*A*), (*G*) Mann-Whitney U-test. *C*, *D*, *E*, *I*, and *J* two-tailed unpaired Student’s *t* test. *H*, two-way ANOVA with Bonferroni *post hoc* test. DMSO, dimethylsulfoxide; DIV, day *in vitro*.

Next, we performed a metabolic analysis focusing on lactate and pyruvate levels, which are metabolites of glycolysis. The results showed that there was no significant difference in lactate levels between the treatment and control groups after axon injury, while pyruvate levels increased earlier in the treatment group (Fig. 4, *B* and *C*). This indicates that glycolysis-derived lactate and pyruvate cannot describe the metabolic mechanism by which harmine promotes axon regeneration. We thus performed RNA-seq analysis to evaluate the impact of harmine on the mitochondrial metabolism of injured cortical neurons. A Venn diagram of gene expression and a Kyoto Encyclopedia of Genes and Genomes enrichment analysis bubble chart of differentially upregulated genes in harmine-treated injured neurons after 4 h were examined. Results showed that harmine significantly upregulated gene expression in the mitochondrial oxidative phosphorylation, suggesting a role as a metabolic driver (Fig. 3, *H* and *I*). Taken together, these findings indicate that harmine’s ability to enhance glucose metabolism and enhance mitochondrial function promotes axon regeneration.

**Figure S5:**
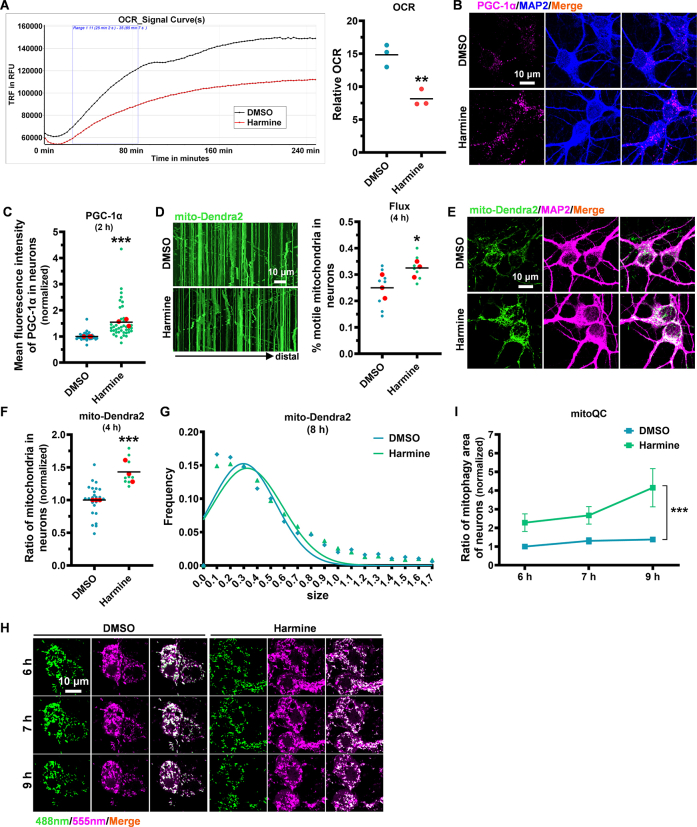


To further characterize the effects of harmine on mitochondrial quality control, we checked several mitochondrial indicators under baseline (uninjured) neuronal conditions. These included neuronal oxygen consumption rate (OCR), mitochondrial biogenesis, transport, and mitophagy. Using time-resolved fluorescence imaging, we observed a decrease in neuronal OCR within 1 h after harmine treatment, indicating that mitochondrial respiration is initially inhibited (Fig. S5*A*). Interestingly, the expression of PGC-1α significantly increased after 2 h of harmine treatment, indicating that harmine facilitates mitochondrial biogenesis (Fig. S5, *B* and *C*).

Next, we observed the behavioral characteristics of neuronal mitochondria following treatment with harmine. We assessed mitochondrial behavior by measuring axonal mitochondrial transport, an indicator of mitochondrial health, using a microfluidic device. We found that mitochondrial transport within axons was enhanced 4 h after treatment (Fig. S5*D*). Previous research suggests that under mild stress, damaged mitochondria are transported to the soma for mitophagy, while healthy mitochondria move to axon terminals to replace aged ones (35). Consistent with this, our findings revealed massive increase in the mitochondrial fraction on the soma-side of the microfluidic device, indicating enhanced mitochondrial turnover within the axons (Fig. S5, *E* and *F*). To precisely quantify the distribution characteristics of mitochondrial size, we performed Gaussian fitting curve analysis. The Gaussian fitting curve for mitochondrial size shifted toward larger values at 8 h post treatment, suggesting the removal of damaged mitochondria (Fig. S5*G*). To determine whether harmine facilitates the clearance of damaged mitochondria through mitophagy, we applied a fluorescent probe, mitoQC, to monitor mitophagy activity at various time points following harmine treatment. We observed a gradual increase in mitophagy levels over time post-harmine treatment (Fig. S5, *H* and *I*). Combining the results of significant increases in ΔΨ_m_ and total ATP levels observed with prolonged harmine treatment (Fig. 2, *M* and *N*), we conclude that enhanced mitochondrial quality control improves mitochondrial and neuronal functions. Taken together, harmine promotes axon regeneration by enhancing glucose metabolism coupled with mitochondrial function.

### Harmine enhances axonal regenerative capacity and functional recovery in mature neurons

To understand the role of mitochondria function in neuronal energy supply, we evaluated the mitochondrial state of neurons at different developmental stages. We found that, compared to young neurons, the mitochondrial load significantly increased in mature neurons (DIV15-DIV30) (Fig. S6*A*). However, as maturation progresses, ATP levels in mitochondria decrease, which is consistent with a reduction in neuronal regenerative capacity (Fig. S6*B*). This finding underscores the importance of enhancing mitochondrial function to activate axon regeneration in mature neurons.

Considering that harmine might enhance glycolytic-mitochondria coupling more effectively in young neurons, leading to higher mitochondrial ATP production, we hypothesized that strengthening this coupling could enhance axonal regeneration in mature neurons that are difficult to regenerate. To test this, we first examined glucose uptake in mature neurons after harmine treatment. As expected, harmine significantly increased glucose uptake (Fig. 4*A*; S6*C*). To determine whether harmine has the ability to maintain mitochondrial quality control in mature neurons, we evaluated mitophagy and mitochondrial transport. Harmine promoted mitophagy in mature neurons and also enhanced mitochondrial transport, suggesting that it had the ability to relocate mitochondria to energy-demanding regions, potentially supporting axon regeneration (Fig. 4, *B* and *C*; S6*D*). As a result, harmine significantly increased the utilization of pyruvate, an intermediate product of glucose metabolism, in mature neurons, without affecting lactate levels and the ECAR (Fig. 4, *D* and *E*; S6*E*). Given that the aforementioned young neurons possess inherent regenerative capabilities, we sought to determine whether harmine remains effective on mature neurons with diminishing endogenous regenerative capacity, specifically those cultured from DIV15 to DIV30. By focusing on these mature neurons, we aimed to isolate the effect of harmine without the confounding influence of inherent regeneration. We confirmed that harmine restored axon regeneration in mature neurons, whereas axons in the control group degenerated (Fig. 4, *F* and *G*). Thus, harmine promotes axon regeneration in mature neurons by enhancing glycolysis couples with mitochondrial function.

To validate the regenerative efficacy of harmine *in vivo*, we used a bilateral sciatic nerve crush model to assess its impact on axon regeneration. As previously described, SCG10 served as a marker to specifically identify regenerated sensory axons within the sciatic nerve (36, 37). We found that harmine significantly upregulated SCG10 expression at the injury site in adult mice, confirming its ability to enhance nerve regeneration (Fig. 4*H*). Moreover, the harmine-treated group exhibited more organized nerve fibers and greater tissue repair in the damaged region (Fig. 4*H*). As the injured sciatic nerve comprises both DRG sensory neurons and motor neurons originating from the spinal cord. We performed a balance beam test to assess the effect of harmine on motor neuron functional recovery. The harmine group exhibited a significantly reduced traversal time on the beam, reflecting improved motor coordination and balance compared to the control group (Fig. 4*I*). Furthermore, the number of slips during walking was notably decreased, and the error rate was significantly lower, indicating that harmine markedly enhances motor function and balance control (Fig. 4*J*). These findings provide additional evidence supporting the effectiveness of harmine in fostering functional recovery in mice. Taken together, harmine has demonstrated considerable neuron repair and functional recovery capabilities *in vivo*.

## Discussion

Glycolysis is closely linked with mitochondrial function to meet cellular energy demands and maintain homeostasis. Normally, glycolysis produces pyruvate, which enters the mitochondria for oxidative phosphorylation, resulting in the efficient production of ATP crucial for cell growth and proliferation (38). However, this coupling can be modulated by various physiological and pathological conditions to enhance energy production. In cancer, this coupling often becomes deregulated. A number of tumor cells exhibit the Warburg effect—elevated glycolysis and disrupted mitochondrial dynamics—resulting in impaired glycolytic-mitochondrial coupling (39). However, recent research presents contradictory reports that the regulation of glycolytic-mitochondrial coupling in tumor cells involves complex mechanisms influenced by genetic and epigenetic changes, with oncogenic signaling pathways often upregulating glycolytic enzymes and modifying mitochondrial function to promote cell growth and survival, leading to speculation that the coupling may be a key regulatory node for cell growth (40, 41).

Our study aimed to investigate whether harmine can promote axon regeneration. Previous research has indicated that harmine promotes periodontal ligament cell-induced tissue regeneration, but its effects and cellular mechanism on axon regeneration have not been explored (19). In our study, we showed that harmine promotes axon regeneration in cultured young neurons and proposed that metabolic coupling is a critical regulatory target (Fig. 4*K*). We demonstrate that harmine supports metabolic coupling by enhancing glucose metabolism, mitophagy, and subsequent mitochondrial biogenesis. Notably, in mature neurons with lower regeneration potential, this strengthened coupling significantly promotes axon regeneration and neuronal repair.

As a small molecule drug, harmine offers several advantages over ID2 overexpression in promoting axon regeneration. Harmine has excellent cell permeability, enabling it to effectively enter neurons and activate their bioactivity (42). In contrast, ID2 overexpression is relatively complicated to implement. Harmine’s dosage can be precisely adjusted for various experimental or therapeutic needs, whereas controlling ID2 overexpression presents more challenges. Additionally, harmine has lower immunogenicity, reducing the risk of immune responses. Collectively, these factors make harmine a more feasible option for treating neuronal injuries compared to ID2 overexpression.

Harmine plays a critical role in neuronal development and regeneration (Fig. 1 and S1). Previous studies have shown that harmine interferes with neurogenesis in cultured hippocampal neurons by inhibiting DYRK1A phosphorylation (21). Our study also found that harmine could reduce both the number and complexity of neuronal neurites (Fig. 1, *D*–*H*). Further investigations revealed that harmine induces neurons to dedifferentiate into an immature state during early development (Fig. S1*G*). Additionally, harmine has been shown to significantly reduce brain edema and hippocampal neuron apoptosis following traumatic brain injury (20). Therefore, harmine not only revives neuron development but also exhibits neuroprotective effects, suggesting its potential as a drug to enhance the intrinsic regenerative capacity of injured neuronal axons.

In the context of neuronal regeneration, intrinsic regenerative capacity may be determined by converting differentiated cells into a more plastic, regenerative state. Notably, increased glycolysis and temporary mitochondrial inhibition could initiate this remodeling process (43). Under normal conditions, harmine enhances mitophagy by coordinating OCR and mitochondrial respiration inhibition, thereby clearing damaged mitochondria. Although enhanced mitophagy was observed with harmine treatment in the first place, it also triggers mitochondrial biogenesis in neurons (Fig. S5*C*), echoing a balanced mutual regulation (44). Thus, harmine maintains mitochondrial function to provide essential energy support for nerve regeneration, making it an effective drug for promoting neuronal axon regeneration.

## Experimental procedures

### Animals

The C57BL/6J mice and Sprague-Dawley rats were purchased from SPF (Beijing) Biotechnology Co., Ltd. The C57BL/6J mice were kept in cages with a 12 h light/dark cycle, and had free access to food and water.

### Reagents and antibodies

The reagents used include harmine (Med Chem Express, Cat# HY-N0737A), MitoTracker Orange CMTMRos (Invitrogen, Cat# M7510), and the Fluorescence Oxygen Sensing Probe (MitoXpress-Xtra, Luxcel Bioscience, Cat# MX-200) and Extracellular Acidification Assay Kit (pH-Xtra, Luxcel Bioscience, Cat# PH200). The anti-βIII tubulin (dilution 1:2000, Cat# G7121, RRID: AB_430874) antibody was purchased from Promega, and the anti-MAP2 (dilution 1:800, Cat# AB5622, RRID: AB_2800501) antibody was obtained from Millipore. The mouse anti-ID2 (dilution 1:500, Cat# sc-398104, RRID:AB_2943636) and anti-PGC-1α (dilution 1:500, Cat# sc-518025, RRID: AB_2890187) antibodies were sourced from Santa Cruz Biotechnology. The anti-Tuj1 (dilution 1:1000, Cat# MMS-435P, RRID:AB_2313773) antibody was acquired from Covance. The anti-SCG10 (dilution 1:3000, Cat# NBP1-49461, RRID: AB_10011569) antibody was procured from Novus. Alexa Fluor 488 goat anti-mouse (dilution 1:1000, Invitrogen, Cat# A-21042, RRID: AB_141357) and Alexa Fluor 555 goat anti-rabbit (dilution 1:1000, Invitrogen, Cat# A-21429, RRID: AB_2535850) were purchased from Invitrogen.

### Preparation of microfluidic devices

We used microfluidic devices that were fabricated in-house through replication of a master mold using polydimethylsiloxane (PDMS, Dow Corning, Cat# DC184). Specifically, the PDMS was purchased from Dow Corning, and its main agent and curing agent were thoroughly mixed at a ratio of 10:1. The mixed PDMS solution was then poured into a predesigned microfluidic mold for replication. Subsequently, the mold containing the PDMS was cured at 80 °C for 120 min. After curing, the PDMS was demolded, and holes were punched at specified locations to accommodate the storage of neuronal basal medium. Finally, the microfluidic device was sterilized for experimental use.

### Cortical neuron culture and establishment of nerve injury model

Before seeding cortical neurons, a sterile 25 mm diameter circular coverslip was placed in a 35 mm culture dish on a clean bench. The coverslip was coated with 2 ml of a mixture of poly-D-lysine hydrobromide (100 μg/ml, Sigma-Aldrich, Cat# P6407) and laminin (10 μg/ml, Roche, Cat# 11243217001), and incubated in a 37 °C, 5% CO_2_ incubator for 2 h. Sprague-Dawley rats no older than 12 h were dissected, their brains isolated, and the meninges removed using curved forceps under a microscope. The cortical neural tissue was cut using curved forceps, minced, and placed in a 15 ml centrifuge tube (45). Mixed 5 ml of Hank’s balanced salt solution (Gibco, Cat# 14025092) buffer into a tube that holds 120 U of papain (Worthington, Cat# LS003127). The tissue in the centrifuge tube was gently pipetted to mix, and then incubated at 37 °C in a 5% CO_2_ incubator for 40 min. The mixture was homogenized by pipetting with a 5 ml pipette, then centrifuged at 300 rpm for 5 min. After centrifugation, the supernatant was discarded; the cells were resuspended in 5 ml of a solution containing protease inhibitors, and allowed to stand for 5 min. The mixture was centrifuged at 300 rpm for 10 min, the supernatant discarded. Preparation of the neuronal basal medium: Neurobasal-A medium (Gibco, Cat# 10888022) at 95.75% (v/v); B27 supplement (Gibco, Cat# 17504044) at 2% (v/v); GlutaMAX supplement (Gibco, Cat# 35050061) at 0.25% (v/v); fetal bovine serum (Gibco, Cat# 10099–141) at 1% (v/v); and penicillin-streptomycin (Gibco, Cat# 15140–122) at 1% (v/v). The cells were resuspended in the neuronal basal medium to obtain the primary neuron cell suspension. A total of 4 × 10^4^ cells per culture dish were seeded into the microfluidic devices. Cortical neuron basal medium was added and incubate at 37 °C in a 5% CO_2_ incubator for 7 days.

The microfluidic device separated the cell bodies and axons of cortical neurons (46). After 7 days of culture, the axons of cortical neurons grew through the microchannel of the microfluidic device to the opposite chamber. The axons were physically severed using a vacuum pump, and a basal medium containing DMSO (Sigma-Aldrich, Cat# D5879) or harmine was added (47). The culture was continued for an appropriate duration. The axons were stained with βIII-tubulin (dilution 1:2000, Promega, Cat# G7121, RRID:AB_430874), imaged under consistent conditions using an Andor spinning disk confocal microscope (Andor, Dragonfly) equipped with a 10 × objective, and axon regeneration was analyzed. ImageJ (RRID:SCR_003070, https://imagej.net/) was used to measure the axon length (μm) or area (μm^2^) of βIII-tubulin fluorescence signal at the same threshold within a 2048 × 2048-pixel. The microscopy image files were loaded into ImageJ and the contrast was adjusted using the “setMinAndMax” function. To quantify axon length, the image was converted to 8 bit format. Then, in the “Measurements” panel, the length parameter was selected, and the NeuronJ plugin was used to track the longest axons in specific regions to obtain the measurement results. For the quantification of the area of regenerating axons, the “Threshold” function was used to extract clear axonal structures, and the “Create Selection” function was used to isolate the regions of interest. The “Set Measurements” command was used to set area measurement parameters, and then the “Measure” command was used to obtain the quantified area of regenerating axons. Images from at least three independent experiments conducted in microfluidic chambers were analyzed.

### Sciatic nerve injury model and treatment

After anesthetizing the male mice (aged 8 weeks) with isoflurane using an anesthesia machine, the mode was switched to mask mode, and the mice were removed from the chamber and placed on the surgical board, with their limbs secured. Curved forceps were used to compress both sides of the exposed nerve at the midsection three times, each for 10 s, and the injury site was marked with a suture knot. Subsequently, the skin was sutured, and the mice were placed in a cage, allowing them to move freely and have unrestricted access to food and water. The mice were randomly divided into a control group and a harmine treatment group, and blinding was not used for intraperitoneal injections. The mice in the treatment group received daily intraperitoneal injections of harmine (25 mg/kg, MedChemExpress, Cat# HY-N0737A). The control group was injected with DMSO after the compression injury.

### Immunofluorescence staining

Immunofluorescence staining was performed on neurons cultured on coverslips. The neurons were fixed with 4% paraformaldehyde (Sigma-Aldrich, Cat# P6148) for 15 min, and then washed three times with PBS. To assess neuronal survival and overall integrity, rabbit anti-MAP2 (dilution 1:800, Millipore, Cat# AB5622, RRID: AB_2800501) antibody was used. For evaluating the regenerative capacity of neuronal axons, mouse anti-βIII-tubulin (dilution 1:2000, Promega, Cat# G7121, RRID: AB_430874) antibodies were used. The following primary antibodies were also used in this study: mouse anti-ID2 (dilution 1:500, Santa Cruz Biotechnology, Cat# sc-398104, RRID: AB_2943636), mouse anti-Tuj1 (dilution 1:1000, Covance, Cat# MMS-435P, RRID: AB_2313773), rabbit anti-SCG10 (dilution 1:3000, Novus, Cat# NBP1-49461, RRID: AB_10011569), mouse anti-PGC-1α (dilution 1:500, Santa Cruz Biotechnology, Cat# sc-518025, RRID: AB_2890187). The neurons were incubated with primary antibodies overnight at 4 °C and wash three times with PBS. Subsequently, Alexa Fluor 488 goat anti-mouse secondary antibody (dilution 1:1000, Invitrogen, Cat# A-21042, RRID: AB_141357) or Alexa Fluor 555 goat anti-rabbit secondary antibody (dilution 1:1000, Invitrogen, Cat# A-21429, RRID: AB_2535850) was added, followed by incubation at room temperature for 1 h. After washing three times with PBS, the coverslips were mounted, and the neurons were observed under a fluorescence confocal microscope.

### Immunohistochemistry

Fix tissue sections with 4% paraformaldehyde and wash twice with PBS for 3 min each. Immerse the fixed tissue in a 20% sucrose solution and incubate at 4 °C for 1 to 2 days until the tissue sinks to the bottom. Transfer the tissue to a 30% sucrose solution and continue incubating at 4 °C until it sinks again. Rinse the tissue with PBS to remove excess sucrose before sectioning. Embed the tissue in an embedding medium and sectioned at a thickness of 20 μm with a freeze-sectioning machine. Store the sections at −80 °C. After thawing the frozen slides at room temperature, wash the sections with PBS. Block nonspecific binding sites by incubating the sections with a blocking solution containing normal serum for 30 min. Following the removal of the blocking solution, add primary antibodies against SCG10 (dilution 1:3000, Cat# NBP1-49461, RRID: AB_10011569), Tuj1 (dilution 1:1000, Cat# MMS-435P, RRID: AB_2313773), and incubate the sections overnight at 4 °C. On the second day, the sections were washed three times with PBS for 5 min each time. Then, Alexa Fluor-conjugated secondary antibodies were added and incubated at room temperature in the dark for 2 h. Sections were washed three times with PBS for 5 min. Finally, sections are mounted with 4′,6-diamidino-2-phenylindole (DAPI) containing mounting medium (Mei5bio, Cat# MF681-plus-01) and stored in the dark at 4 °C. Images were randomly captured using an Andor spinning disk confocal microscope.

### Neuronal dendrite complexity analysis

Images of MAP2-stained neurons were acquired with an Andor spinning disk confocal microscope. Sholl analysis was conducted with the “Sholl analysis” plugin in ImageJ. Dendritic complexity was assessed using concentric circles (10 μm intervals) centered at the manually defined soma, with the maximum radius corresponding to the longest dendrite length. The Sholl plugin calculated the number of dendrite intersections at each concentric circle. Data from different treatment groups (DMSO, DIV0, DIV2, and DIV3) were statistically analyzed using GraphPad Prism (RRID:SCR_002798; http://www.graphpad.com/) to determine the mean dendrite intersections and the number of intersections at different distances from the soma.

### Glucose uptake assay

We utilized a glucose assay kit and UP02 staining (dilution 1:1000, Dojindo, Cat# UP02-1set) to measure glucose uptake in neurons. According to the manufacturer’s protocol for the glucose assay kit, the homogenized sample solution was first prepared. Subsequently, the sample was added to the enzymatic reaction system. The resulting color change from the enzymatic reaction was proportional to the glucose content and was quantified by measuring the absorbance at a specific wavelength (540 nm) using CLARIOstarPlus-ACU analyzer (BMG LABTECH).

For detecting glucose uptake in neurons using UP02 staining, the UP02 fluorescent probe was added to a prewarmed culture medium, and the neurons were incubated with the probe for 15 min. After the incubation period, the neurons were washed with wash and incubation solution to remove any unbound UP02. Subsequently, the fluorescence intensity of UP02 in the neurons was detected using a confocal fluorescence microscope. Finally, the mean fluorescence intensity was calculated using ImageJ software. Specifically, the contrast was adjusted using the “setMinAndMax” function to display the cellular fluorescence signals. Then, the “Threshold” function was applied to extract clear neuron structures, and the Create Selection feature was utilized to isolate the regions of interest. Measurement parameters were set using the “Set Measurements” command, including Mean gray value, integrated density, Area fraction, Area, and Limit to threshold. Finally, the “Measure” command was used to obtain the mean fluorescence intensity of the UP02 fluorescent probe in the neurons.

### Measurement of mito-Dendra2, iSnFR-ATP, mitoQC, Laconic, and Pyronic, mitoGoAteam

Cortical neurons from both the control and harmine-treated groups were infected with lentiviruses encoding mitoQC (Addgene, Cat# 78520, RRID: Addgene_78520), Laconic (Addgene, Cat# 44238) and Pyronic (Cat# 51308, RRID: Addgene_51308) at DIV0. The mito-Dendra2, mitoGoAteam, and iSnFR-ATP plasmids were synthesized and constructed in our own laboratory. Plasmid DNA sequences can be provided upon request. At DIV7, axotomy was performed on the neurons, and 10 μM harmine was added to the treatment group. Live-cell imaging was conducted 4 h post treatment, with imaging regions randomly selected. For imaging mito-Dendra2 and iSnFR-ATP probes, excitation was performed at 488 nm, and emission was detected at 521 nm (48, 49). mitoQC was excited at 488 nm and 555 nm, with emission collected at 521 nm and 594 nm (50). Laconic and Pyronic were excited at 440 nm, with emission detected at 488 nm and 521 nm, respectively (51, 52). The mitoGoAteam was excited at 488 nm, with emission detected at 510 nm and 560 nm, respectively (53). Pseudo-color images and ratios were calculated using ImageJ software. We first used the “setMinAndMax” function to adjust the contrast range of each channel. The “Threshold” function was used to extract clear neuronal structures, and the Create Selection function was used to isolate regions of interest. The “Set Measurements” command configured measurement parameters, and the “Measure” function measured the mean fluorescence intensity of each channel. To obtain the fluorescence ratio image, the “Split Channels” function separated images of different emission wavelengths, followed by using the “Image Calculator” to compute and generate ratio images. The “Spectrum” function was run to add pseudocolor and a Calibration Bar. Finally, the ratio image was converted to RGB format, with the background color adjusted to black.

### Lentivirus preparation and infection of cortical neurons

The HEK293T cells (American Type Culture Collection (ATCC), Cat# CRL-3216, RRID:CVCL_0063) were obtained from the ATCC, verified for authenticity, tested for quality, and maintained following the guidelines provided by ATCC. All cells have undergone short tandem repeat profiling for authentication and have been confirmed to be free of *mycoplasma*. Lentiviruses encoding mito-Dendra2, iSnFR-ATP, mitoQC, Laconic, Pyronic, and mitoGoAteam were produced by transfecting HEK293T cells. The detailed procedures for lentivirus particle production, purification, and infection were described as follows (54). In brief, HEK293T cells used for lentivirus production were cultured in Dulbecco’s modified eagle medium (Hyclone, Cat# SH30243.01) containing 10% fetal bovine serum (Gibco, Cat# 10099–141) and 0.5 mM GlutaMAX (Gibco, Cat# 35050061), and were transfected with the target vectors psPAX2 (Addgene, Cat# 12260, RRID: Addgene_12260) and pMD2.G (Addgene, Cat# 12259, RRID: Addgene_12259) in a 2:2:1 ratio. At 16 h post transfection, the medium was replaced with UltraCULTURE medium (Lonza, Cat# BEBP12–725F) supplemented with 1% sodium pyruvate (Gibco, Cat# 11360070), 1% sodium bicarbonate (Gibco, Cat# 25080094), 1% GlutaMAX (Gibco, Cat# 35050061), and 10 mM Hepes (Gibco, Cat# 15630080). The viral supernatant was collected at 24 h and 48 h post medium change, followed by filtration through a 0.45 μm filter (Millipore, SLHV033RB). The filtered viral supernatant was then centrifuged at 34,000 rpm for 120 min at 4 °C. The concentrated viral particles were resuspended in 80 μl of precooled viral preservation solution, aliquoted, and stored at −80 °C until use. For infection of cortical neurons, 1 to 2 μl of the concentrated lentivirus was added to 1 × 10^5^ cortical neurons, depending on the vector size.

### Measurement of mitochondrial membrane potential (ΔΨ_m_)

To measure the mitochondrial membrane potential, neurons were incubated with 50 nM MitoTracker Orange CMTMRos probe (Invitrogen, Cat# M7510) at 37 °C for 30 min. This probe passively diffused across the cell membrane and accumulated in active mitochondria, with the extent of accumulation being dependent on the mitochondrial membrane potential (1, 55). After incubation, the medium was replaced with Live Cell Imaging Solution (Invitrogen, Cat# A14291DJ), and live cell images were collected using an Andor spinning disk confocal microscope. Regions for image acquisition were randomly selected.

### Time-lapse imaging of live neurons and kymograph analysis

To visualize mitochondrial movement within axons, the neuronal base medium in the microfluidic devices was replaced with Live Cell Imaging Solution (Invitrogen, Cat# A14291DJ). Live-cell time-lapse imaging was then performed using an Andor spinning disk confocal microscope, with images captured every 5 s for a total of 100 consecutive frames. Real-time analysis of mitochondrial transport kinetics was conducted using ImageJ software (56). In ImageJ, the “Kymograph” plugin was selected. The “Width Filament/wide Line” was set to 50 pixels in the generated image, and a line was drawn along the path of mitochondrial movement. The “Straighten” function was then used to generate the straightened filament image. Subsequently, “Stack” was selected, and “OK” was clicked to produce the kymograph image. Mitochondria that remained stationary throughout the recording period were classified as static, while those that moved had a net displacement greater than 5 μm.

### Measurement of extracellular OCR and ECAR

Time-resolved fluorescence was employed using MitoXpress-Xtra (Luxcel Bioscience, Cat# MX-200) and pH-Xtra probes (Luxcel Bioscience, Cat# PH200) to measure the OCR and ECAR of neurons. This approach detected wavelength and time parameters simultaneously, eliminating nonspecific fluorescence interference and significantly improving analytical sensitivity. MitoXpress-Xtra, an oxygen-sensitive fluorescent probe, calculated OCR by measuring fluorescence intensity or lifetime (57). Cortical neurons were seeded in 96-well plates (5 × 10^4^ cells/well) and incubated for 7 days. After this period, cells were treated with either DMSO (control group) or harmine (treated group) and incubated for an additional 48 h. The neuronal culture medium in each well was replaced with 150 μl of fresh, prewarmed medium at 37 °C, and the plate was equilibrated in a heated incubator at 37 °C. Subsequently, 10 μl of OCR probe solution (1 μM) was added to each well, followed by 10 μM harmine dissolved in DMSO (only DMSO was added to the control wells). Immediately after, 100 μl of prewarmed mineral oil was added to each well. The prepared samples were then placed in a prewarmed CLARIOstarPlus-ACU analyzer at 37 °C, and the preset OCR-Xtra analysis protocol was selected. Oxygen consumption was monitored over a period of 240 min. Using the CLARIOstar-Data Analysis software (https://www.bmglabtech.cn/microplate-reader-software/), the OCR signal curves were obtained, and the OCR values were calculated based on the slope within the selected time range.

Unlike OCR measurement, ECAR detection involved placing cells in a CO_2_-free incubator to remove residual CO_2_ before starting the assay. Prior to the experiment, a vial of probe powder was reconstituted in 1 ml of prewarmed buffer at 37 °C. The old culture medium was removed from the neurons in the 96-well plate, and the wells were washed twice with 100 μl of buffer. Then, 150 μl of respiration buffer was added to each well, and the plate was equilibrated in a heated incubator at 37 °C. Subsequently, 10 μl of the reconstituted probe (dilution 1:160) and 10 μM harmine were added to each well and mixed thoroughly. The prepared samples were placed in a prewarmed ClariostarPlus-ACU analyzer at 37 °C, and the preset ECAR analysis protocol was selected. Extracellular acidification was monitored over a period of 240 min. Using the CLARIOstar Data Analysis software, the ECAR signal curves were obtained and the ECAR values were calculated based on the slope within the selected time range.

### RNA-seq analysis

Cortical neurons (5 × 10^6^ cells) were seeded into large-scale microfluidic system devices and cultured in 10 cm dishes for 7 days. At DIV7, axotomy was performed on the neurons, followed by treatment with or without harmine (10 μM). Total RNA was extracted using the RNA extraction kit (TIANGEN, Cat# dp430) according to the manufacturer’s instructions 4 h post treatment. The concentration and quality of the extracted RNA were assessed using a NanoDrop 2000 spectrophotometer (Thermo Fisher Scientific). Further quality assessment of the RNA was performed using an Agilent bioanalyzer (Agilent Technologies), and sequencing was carried out by BGI (Beijing Genomics Institute). Differentially expressed genes identified from the sequencing data were subjected to bioinformatics analysis.

### Behavioral evaluation

The beam walking task was used to assess the balance ability of mice. The beam had a diameter of approximately 20 mm, a total length of 90 cm, and a height of 30 cm. The ends of the beam were connected to a starting platform and a target box, respectively. Mice were placed on the starting platform, and the latency to reach the target box, along with the number of sideslip errors made during the walk, was recorded to characterize their recovery of motor function.

### Statistical analyses

Statistical analyses were performed using GraphPad Prism version 8.4.3. There were no exclusion criteria, all experimental mice underwent bilateral sciatic nerve injury, and all experimental data were included in the analysis. Blinding was not used in the experiment. Instead, we followed the principle of randomization, randomly allocated the animals to experimental groups. We randomly selected areas for microscopic image acquisition in each biological replicate. In this study, biological replicate experiments (N) were conducted with independent samples from at least three different biological individuals, based on our previous experience. Each biological replicate experiment was accompanied by technical replicate experiments (n), which involved multiple measurements or experimental operations on the same biological sample. We used GraphPad Prism to perform normality and lognormality tests. All data were analyzed using either unpaired *t* test or two-way ANOVA, and the results were presented as the mean or mean ± SEM. Values of *p* < 0.05 were regarded as indicating statistical significance (∗*p* < 0.05; ∗∗*p* < 0.01; ∗∗∗*p* < 0.001; ns, not significant).

## Data availability

The data are available in the article and its supplementary materials. Original data from this study can be obtained upon request from the corresponding author.

## Supporting information

This article contains supporting information.

## Ethics statement

All animal experiments were approved by the Ethics Committee of Beihang University (approval code: BM20210060) and were conducted in accordance with its guidelines.

## Conflict of interest

The authors declare that they have no conflicts of interest with the contents of this article.

## Figures and Tables

**Figure S6:**