# Supplementary material for: Harmine promotes axon regeneration through enhancing glucose metabolism
Source: J Biol Chem. 2025 Feb 2;301(3):108254. doi: 10.1016/j.jbc.2025.108254 (PMC11927705; doi:10.1016/j.jbc.2025.108254)
Supplement: Supporting Information [file mmc1.docx]

Supporting Information for

**Harmine promotes axon regeneration through enhancing glucose metabolism**

Ruixuan Liu^1, 3^, Bing Zhou^1, 2,^*

^1^Beijing Advanced Innovation Center for Big Data-Based Precision Medicine, Beihang University, Beijing 100191, China;

^2^Interdisciplinary Innovation Institute of Medicine and Engineering Interdisciplinary, Beihang University, Beijing 100191, China;

^3^School of Biological Science and Medical Engineering, Beihang University, Beijing 100191, China

* Correspondence: zhoub2@hotmail.com

**This file includes:**

Fig. S1 to page S-2

Fig. S2 to page S-3

Fig. S3 to page S-4

Fig. S4 to page S-5

Fig. S5 to page S-6

Fig. S6 to page S-7

### Supporting Information Figures

**Fig. S1. Harmine promotes axon regeneration through the regulation of neuronal branching and reduces neuronal maturity. Related to Fig. 1.**

(A) Cortical neurons were cultured for 3 days and then exposed to different concentrations of harmine (0, 8, 10, 16 μM) for an additional 48 h, followed by βIII-tubulin staining (green). Scale bar, 100 μm. (B) Quantitative analysis of neuronal cell density after treatment with different concentrations of harmine (0, 8, 10, 16 μM) in (A). (0 μM vs 8 μM: *p* = 0.2564; 0 μM vs 10 μM: *p* = 0.0582; 0 μM vs 16 μM: *p* = 0.1458). N_(0 μM)_ = 3, N_(8 μM)_ = 3, N_(10 μM)_ = 3, N_(16 μM)_ = 3. (C) Quantitative analysis of total neurite length after treatment with different concentrations of harmine (0, 8, 10, 16 μM) in (A). (0 μM vs 8 μM: *p* = 0.0148; 0 μM vs 10 μM: *p* = 0.0022; 0 μM vs 16 μM: *p* = 0.0041). N_(0 μM)_ = 3, N_(8 μM)_ = 3, N_(10 μM)_ = 3, N_(16 μM)_ = 3. (D) Cortical neurons were treated with harmine at DIV0, DIV2, and DIV3, respectively, and then stained with MAP2 (red) at DIV7. Scale bar, 10 μm. (E) Quantitative analysis of neurite number in neurons treated with harmine at DIV0, DIV2, and DIV3, and then cultured to the same time point (DIV7) in (D). (DMSO vs DIV0: *p* < 0.001; DMSO vs DIV2: *p* = 0.0399; DMSO vs DIV3: *p* = 0.0674). N = 3; n_(DMSO)_ = 79, n_(DIV0)_ = 112, n_(DIV2)_ = 108, n_(DIV3)_ = 57. (F) Distribution of neurite number in neurons treated with harmine at DIV0, DIV2, and DIV3, and then cultured to the same time point (DIV7) in (D). (DMSO vs DIV0: *p* < 0.001; DMSO vs DIV2: *p* = 0.0025; DMSO vs DIV3: *p* = 0.9998). N_(DMSO)_ = 3, N_(DIV0)_ = 3, N_(DIV2)_ = 3, N_(DIV3)_ = 3. (G) The mean dendrite intersections were measured by means of Sholl analysis in (D). (DMSO vs DIV0: *p* < 0.001; DMSO vs DIV2: *p* < 0.001; DMSO vs DIV3: *p < 0.001*). N = 3; n_(DMSO)_ = 65, n_(DIV0)_ = 70, n_(DIV2)_ = 81, n_(DIV3)_ = 60. (H) Sholl analysis of dendritic branching in (D) demonstrated that harmine significantly reduces dendritic complexity during early development. (DMSO vs DIV0: *p* < 0.001; DMSO vs DIV2: *p* < 0.001; DMSO vs DIV3: *p <* 0.001). N = 3; n_(DMSO)_ = 65, n_(DIV0)_ = 70, n_(DIV2)_ = 81, n_(DIV3)_ = 60. (I) Fluorescent images (left) and quantitative analysis (right) of neuronal maturation. DIV3 neurons were treated with DMSO or harmine for 48 h, followed by immunofluorescence staining with MAP2 (cyan) to specifically label dendrites, and βIII-tubulin (magenta) to specifically label axons. (DMSO vs Harmine: *p* = 0.0495). N = 3; n_(DMSO)_ = 28, n_(Harmine)_ = 28. Scale bar, 100 μm. Data in (C), (F) and (H) were expressed as mean ± SEM. Data in (B), (E), (G) and (I) were expressed as mean. Significance: **p* < 0.05, ***p* < 0.01, ****p* < 0.001; ns, not significant. (B), (C), (I) Two-tailed unpaired Student’s t-test. (E), (G) Mann-Whitney U-test. (F), (H) Two-way ANOVA with Dunnett’s post hoc test.

**Fig. S2. Harmine increases pyruvate utilization. Related to Fig. 2.**

(A) Pyruvate utilization increased in neurons after 4 h of harmine treatment, as observed using a pyruvate assay kit. (DMSO vs Harmine: *p* = 0.0487). N_(DMSO)_ = 3, N_(Harmine)_ = 3. (B) Cultured cortical neurons were treated with DMSO or harmine for the corresponding time point. ECAR was measured at predetermined time points using pH-Xtra (left). Using the ClariostarPlus-ACU analyzer, ECAR was quantified by calculating the slope over a selected time range (right). (DMSO vs Harmine: *p* = 0.4541). N_(DMSO)_ = 3, N_(Harmine)_ = 3. Data were expressed as mean. Significance: **p* < 0.05; ns, not significant. (A), (B) Two-tailed unpaired Student’s t-test.

**Fig. S3. Harmine regulates ID2 protein expression and metabolic adaptation. Related to Fig. 2.**

(A) RNA-seq analysis of cortical neurons untreated (DMSO) and treated with harmine (Harmine). Yellow indicates upregulated genes; blue indicates downregulated genes after harmine treatment. (B) The heatmap showing significantly differentially expressed genes (DEGs) in cortical neurons after 4 h of harmine treatment from RNA-seq analysis, and their expression in control cortical neurons. Red indicates upregulated genes; blue indicates downregulated genes. (C) Quantitative analysis of increased ID2 expression levels in cortical neurons after 4 h of harmine treatment. (DMSO vs Harmine: *p* = 0.0361). N_(DMSO)_ = 3; N_(Harmine)_ = 3. (D) Representative images of cortical neurons treated with harmine. ID2 (green), MAP2 (magenta). Scale bar, 10 μm. (E) Effect of different concentrations of harmine on ID2 protein expression. The graph shows the quantitative analysis of ID2 protein expression after 0, 8, 10, and 20 μM harmine treatment in (D), normalized to the control group. (0 μM vs 2.5 μM: *p* = 0.0217; 0 μM vs 5 μM: *p* < 0.001; 0 μM vs 10 μM: *p* = 0.0009; 0 μM vs 20 μM: *p* = 0.0254). N = 3; n_(0 μM)_ = 29, n_(2.5 μM)_ = 27, n_(__5 μM)_ = 28, n_(10 μM)_ = 31, n_(20 μM)_ = 21. Data were expressed as mean. Significance: **p* < 0.05, ****p* < 0.001. (C), (E) Two-tailed unpaired Student’s t-test.

**Fig. S4. Harmine promotes axon regeneration by regulating glucose metabolism and mitochondrial function. Related to Fig. 3.**

(A) Fluorescent images (left) and quantitative analysis (right) of PGC-1α protein expression in DIV7 cortical neurons treated with or without harmine for 2 h after axotomy. (DMSO_Ax vs Harmine_Ax: *p* < 0.001). N = 3; n_(DMSO_Ax)_ = 40, n_(Harmine_Ax)_ = 36. PGC-1α (magenta), MAP2 (blue). Scale bar, 10 μm. (B) The changes in lactate levels in neurons from the control group (before) and after different durations (4, 8, 48 h) of harmine treatment post-axotomy were measured using ClariostarPlus-ACU analyzer. (DMSO vs Harmine: *p* = 0.1480). N_(DMSO)_ = 3, N_(Harmine)_ = 3. (C) The changes in pyruvate levels in neurons from the control group (before) and after different durations (4, 8, 48 h) of harmine treatment post-axotomy were measured using ClariostarPlus-ACU analyzer. (DMSO vs Harmine: *p* = 0.7616). N_(DMSO)_ = 3, N_(Harmine)_ = 3. Data in (A) were expressed as mean. Data in (B) and (C) were expressed as mean ± SEM. Significance: ns, not significant. (A) Mann-Whitney U-test. (B), (C) Two-way ANOVA with Bonferroni post hoc test.

**Fig. S5. Harmine maintaining mitochondrial homeostasis under baseline condition in neurons. Related to Fig. 3.**

(A) Cultured cortical neurons were treated with DMSO or harmine for 240 min. At the designated time points, the MitoXpress-Xtra probe was added, and the OCR signal curves (left) and the slope within the selected time range were obtained using the ClariostarPlus-ACU analyzer to calculate OCR (right). (DMSO vs Harmine: *p* = 0.0056). N_(DMSO)_ = 3, N_(Harmine)_ = 3. (B) Representative images of PGC-1α immunofluorescence staining of cortical neurons after 2 h of harmine treatment. PGC-1α (magenta), MAP2 (blue). Scale bar, 10 μm. (C) Quantitative analysis of PGC-1α expression in (B) was performed. (DMSO vs Harmine: *p* < 0.001). N = 3; n_(DMSO)_ = 36, n_(Harmine)_ = 39. (D) Kymographs (left) and quantitative analysis (right) showed that axonal mitochondrial activity in neurons increased after harmine treatment. Cortical neurons were infected with lentivirus mito-Dendra2 (green). Time-lapse images were collected at DIV7 recorded for 100 frames at 5 s intervals. In the kymographs, vertical lines represent stationary mitochondria, and arrows indicate the direction of distal mitochondrial movement. (DMSO vs Harmine: *p* = 0.0497). N = 3; n_(DMSO)_ = 8, n_(Harmine)_ = 7. Scale bar, 10 μm. (E and F) Representative images (E) and quantitative analysis (F) of the ratio of neuronal area occupied by mito-Dendra2-labeled mitochondria in control (DMSO) and treated group (Harmine), normalized to the control group. (DMSO vs Harmine: *p* < 0.001). N = 3; n_(DMSO)_ = 24, n_(Harmine)_ = 9. mito-Dendra2 (green), MAP2 (magenta). Scale bar, 10 μm. (G) Quantitative analysis of the Gaussian fitting curves of mitochondrial size distribution labeled with mito-Dendra2 in the control group (DMSO) and the harmine-treated group (Harmine) after axotomy at DIV7. N = 3; n_(DMSO)_ = 18, n_(Harmine)_ = 18. (H and I) Representative images (H) and quantitative analysis (I) of mitophagy in lentiviral mitoQC-infected neurons were observed at different time points (6, 7, 9 h) in the control group (DMSO) and the treated group (Harmine) at DIV7 using fluorescence confocal microscopy. Fluorescent images excited at 488 nm (green) represent healthy mitochondria, while those excited at 555 nm (magenta) represent both healthy mitochondria and autophagic mitochondria. (DMSO vs Harmine: *p* < 0.001). N = 3; n_(DMSO)_ = 10, n_(Harmine)_ = 10. Scale bar, 10 μm. Data in (A), (C), (D), (F) were expressed as mean. Data in (I) were expressed as mean ± SEM. Significance: **p* < 0.05, ***p* < 0.01, ****p* < 0.001. (A), (D), (F) Two-tailed unpaired Student’s t-test. (C), (D) Mann-Whitney U-test. (I) Two-way ANOVA with Bonferroni post hoc test.

**Fig. S6. Harmine regulates glucose metabolism and mitochondrial homeostasis in mature neurons. Related to Fig. 4.**

(A) Representative images showing mitochondrial status in young and mature neurons, with mito-Dendra2 (green) and MAP2 (magenta) staining (left). Quantification of the proportion of mitochondria in young and mature neurons (right). (Young vs Mature: *p* < 0.001). N = 3; n_(Young)_ = 49, n_(Mature)_ = 96. Scale bar, 5 μm. (B) Cortical neurons were infected with lentivirus mitoGoAteam at DIV0 to label mitochondrial ATP (left). Images were pseudo-colored via the ratio of fluorescence emitted at 560 nm and 510 nm. Scale bar, 5 μm. Subsequently, mitochondrial ATP levels in the neurons were measured at DIV5 and DIV18 (right). (Young vs Mature: *p* < 0.001). N = 3; n_(Young)_ = 37, n_(Mature)_ = 31. (C) Glucose uptake increased in mature neurons of both control and harmine-treated groups after axotomy, as observed using a glucose assay kit. (DMSO vs Harmine: *p* = 0.0055). N = 3; n_(DMSO)_ = 9, n_(Harmine)_ = 8. (D) Kymographs (left) and quantitative analysis (right) showed mitochondrial shuttling in axons of mature neurons after harmine treatment. Cortical neurons were infected with lentivirus mito-Dendra2 (green). Time-lapse images were collected at DIV30 recorded for 100 frames at 5 s intervals. In the kymographs, vertical lines represent stationary mitochondria, and arrows indicate the direction of distal mitochondrial movement. (DMSO vs Harmine: *p* = 0.003). N_(DMSO)_ = 5, N_(Harmine)_ = 4. Scale bar, 10 μm. (E) Cultured mature cortical neurons were treated with DMSO or harmine for corresponding time periods. At designated time points, ECAR was measured using pH-Xtra (left). Using the ClariostarPlus-ACU analyzer, ECAR was quantified by calculating the slope over a selected time range (right). (DMSO vs Harmine: *p* = 0.6388). N_(DMSO)_ = 3, N_(Harmine)_ = 3. Data were expressed as mean. Significance: ***p* < 0.01, ****p* < 0.001; ns, not significant. (A), (B), (D), (E) Two-tailed unpaired Student’s t-test. (C) Mann-Whitney U-test.
